# Monitoring of the Physical and Chemical Properties of a Gasoline Engine Oil during Its Usage

**DOI:** 10.1155/2012/819524

**Published:** 2012-03-27

**Authors:** Behnam Rahimi, Abolfazl Semnani, Alireza Nezamzadeh-Ejhieh, Hamid Shakoori Langeroodi, Massoud Hakim Davood

**Affiliations:** ^1^Department of Chemistry, Shahreza Branch, Islamic Azad University, Isfahan, Shasreza 86145-311, Iran; ^2^Department of Chemistry, Faculty of Sciences, University of Shahrekord, Shahrekord 88186-34141, Iran; ^3^R&D Unit, Barzin Sepand Sepahan Company, Dehagh Industrial City, Isfahan 19615-134, Iran; ^4^Quality Control Unit, Sepahan Oil Company, Isfahan 15119-15513, Iran

## Abstract

Physicochemical properties of a mineral-based gasoline engine oil have been monitored at 0, 500, 1000, 2000, 3500, 6000, 8500, and 11500 kilometer of operation. Tracing has been performed by inductively coupled plasma and some other techniques. At each series of measurements, the concentrations of twenty four elements as well as physical properties such as: viscosity at 40 and 100°C; viscosity index; flash point; pour point; specific gravity; color; total acid and base numbers; water content have been determined. The results are indicative of the decreasing trend in concentration of additive elements and increasing in concentration for wear elements. Different trends have been observed for various physical properties. The possible reasons for variations in physical and chemical properties have been discussed.

## 1. Introduction

Oil analysis involves sampling and analyzing oil for various properties and materials to monitor wear and contamination in an engine, transmission, or hydraulic system [1]. Sampling and analyzing on a regular basis establish a baseline of normal wear and can help indicate when abnormal wear or contamination is occurring. Oil Analysis not only provides a window into the mechanical condition of the component, but also determines the condition of the oil itself to help optimize drain periods [2–4].

 The first use of used oil analysis dates back to the early 1940s by the railway companies in the Western United States. Prompted by the purchase of a fleet of new locomotives, technicians used simple spectrographic equipment and physical tests to monitor locomotive engines [5, 6]. As steam locomotives gave yield to diesel locomotives, oil analysis practices by railways were caught on. By the 1980s, oil analysis formed the basis of condition-based maintenance in most railways in North America. Owing to the success of oil analysis in the railways, the American Navy used spectrometric techniques to monitor jet engines on their aircraft in the mid 1950s. Around this time, Rolls-Royce was also experimenting with oil analysis for their jet turbines. Oil analysis began to spread and programs were developed by the American Army and Air Force throughout the 1950s and early 1960s. Then commercial oil analysis laboratories first appeared on the scene in the early 1960s [5, 6].

 At present oil analysis regards an important part of condition monitoring in advanced industrial countries. By employment of such programs, considerable saving in time and costs has been obtained [7, 8]. Beside technical reports, a number of papers with the subject of oil analysis can be found in literature [9–16]. In diverse papers the application of a wide range of analytical procedures and methods such as potentiometry [15], polarography [16], inductively coupled plasma [17], Fourier transformation infrared spectroscopy [18, 19], atomic absorption spectroscopy [20], differential scanning gravimetry [21], X-ray fluorescence spectroscopy [22], laser-induced break down spectroscopy [23], spectrography [24], ferrography [25], mass spectrometry [26], and chromatography [27] have been described.

 In an oil analysis, the concentration of a number of elements as well as the quantity of some of the physical properties such as viscosity, viscosity index, density, flash point, pour point, total acid and base numbers, and water content [28, 29] is determined. The resulting data are then employed for the diagnosis of the conditions of oil and motor [2]. Oil analysis can detect, fuel dilution of lubrication oil, dirt contamination in the oil, antifreeze in the oil, excessive bearing wear, and misapplication of lubricants. Early detection can reduce repair bills, diminish catastrophic failures, increase machinery life, and lessen nonscheduled downtime [2]. 

 We have recently been involved in the investigation of lubrication oils [30–32]. In this paper, we report the results of the physical and chemical monitoring of a mineral-based gasoline lubricant at different operation kilometers. The selected oil is a product from Sepahan Oil Company. The tracing has been performed by ICP-OES and some other techniques.

## 2. Experimental

### 2.1. Materials

 Base oil SN-500 and gasoline oil Speedy SL from Sepahan Oil Company were used directly. Methanol, hydrochloric acid, perchloric acid, different buffers, propane-2-ol, chloroform, potassium hydroxide, acetic acid, acetic anhydride, chlorobenzene, sodium perchlorate, xylene, acetone, and solid carbon dioxide were purchased from Merck Company and used without any processing. Spex multielement primary standards set were used for ICP-OES elemental analysis.

### 2.2. Test Methods

 The test methods were followed as: ASTM D-445 for viscosity at 40°C and 100°C, ASTM D-2270 for viscosity index, ASTM D-92 for flash point, ASTM D-97 for pour point, ASTM D-1298 for specific gravity, ASTM D-1500 for color, ASTM D-664 for total acid number, and ASTM D-6304 for water content.

### 2.3. Instrumental

 All of the viscosities, viscosity indices and specific gravities were determined by viscometer Anton Paar model SVM 3000. Flash points were evaluated by flash point tester Herzog model HC 852. Pour points were determined by pour point tester Herzog model HC 852. The colors were determined by Dr. Long instrument. TBNs were determined by robotic titrosampler Metrohm model Dosiono 800. TANs were determined by titrator Metrohm model Titrino MPT 789. FTIR spectrum was recorded on a FTIR spectrum Perkin Elmer model Spectrum 65 using KBr pellet. The elemental analysis of the base oil, that is, SN-500 and formulated oil (Speedy SL) was performed by ICP-OES Perkin Elmer model Optima 5300 V. The detection limits (DLs) were obtained under simultaneous multielement conditions with the axial of a dual-view plasma using a cylonic spray chamber and a concentric nebulizer. All detection limits are given in microgram per liter and were determined using organometallic standards. The selected wavelengths and the values of DL (values in parentheses) for each element are as shown in Table 4.

### 2.4. Sampling

 At each running kilometer the sampling [33] was performed immediately after turning off the car. Adequate amount of oil sample was taken by 100 mL syringe.

## 3. Results and Discussion

The concentrations of twenty-four element in lubrication oil and at different kilometers have been determined by ICP-OES. The corresponding values are given in Table 1. Also, the standard deviations due to each of the data are given in Table 2. The results have been sorted based on the decreasing trend in fresh oil. At first glance, the obtained data can be divided to three groups, (i) elements that their concentrations are more than 10 ppm, (ii) elements with concentration less than 10 ppm but more than LD, and (iii) elements, which do have below LD concentration. According to this categorization sulfur, zinc, phosphorous, magnesium, silicon, calcium, and barium can be located in the first group, boron, molybdenum, aluminum, silver, chromium, nickel, and sodium are the members of the second group and the rest of elements, that are, manganese, iron, copper, tin, titanium, vanadium, lead, cadmium, antimony, and potassium belong to third group. On the other hand, the obtained data indicate that upon the continuous usage of the oil and at higher kilometers, the concentration of some of the elements decreases continuously, mean while an increasing trend is observed for other elements. Thus, a decreasing trend is observed for the elements no. 1–8 (Table 1) and an increasing trend exists for other elements.

 One of the sources of the elements in fresh oil is additives, that are, the compounds which are employed in the oil formulation and do have the role of enhancement of the physicochemical properties of the oils [34]. Depending on the application, various combinations of additives are used to meet the required performance level; the most important are detergents, dispersants, antiwear, antioxidants, viscosity modifiers, foam inhibitors, and pour point depressants [35, 36]. Thus, zinc dialkyldithiophosphates (ZDDPs) are common antiwear and antioxidant, which contain Zn, P, and S in their structure [37], calcium and barium salts of long chain alkylarylsulfonic acids are common Ca-containing detergents [34], and liquid silicones are the most efficient antifoam agents, which include Si in their structure [34].

 The other source of the elements in the fresh lubricant oil are those elements, which in the process of base oil production are incorporated. Because of the organic character of the base oil, it is anticipated that its metallic elements are less than the nonmetallic ones.

 Thus, the elements of no. 1–14 in fresh oil originate from two sources of base oil and additives. In the case of metallic elements it is anticipated that the base oil has minor contribution and the main part is due to additives. In other cases such as sulfur and phosphorus, the contribution of both sources may be considerable.

 In order to have a better understanding of the sources of the elements in the fresh oil, it was also examined for different elements. The obtained results are given in the second column of Table 1 (values in parenthesis). As it can be seen, except S, the concentration of other elements is less than 10 ppm. Considering that the employed base oil, belongs to group (I), such a high level of S is not abnormal. On the other hand, because of organic character of the base oil the low concentration of metallic elements is not unexpected.

 As the data shown in the fresh oil S does have the most concentration. This can be attributed to (i) high level of S in the base oil and (ii) application of ZDDP, which is a sulfur-containing additive and normally is employed in crankcase oil formulations.

 Zinc and phosphorous are the second and third highest concentration elements (Table 1). The comparison of the level of these elements in fresh oil with those of base oil indicates huge increase in the later relative to the former. Such an observation can also be assigned to the usage of ZDDP (as a Zn and P containing additive) in the oil formulation.

 The elevated levels of Mg, Si, Ca, and Ba can be related to the application of additives such as basic phenates or magnesium sulfonate, silicon antifoam, calcium sulfonate, and barium sulfonate [34].

 Comparison of the concentrations of the elements no. 7–14 in the base oil with those of fresh oil (Table 1) does not show any significant change. Therefore, these elements are merely originated from the base oil.

 None of the elements no. 16–24 (Table 1) exist in the base oil. Also they cannot be found in fresh oil. This means that in the lubricant formulation, additives which contain the recent elements have not been employed.

 The concentrations of elements no. 1 to 7, which are incorporated in additive structures, versus the running kilometer are given in Table 1. As it can be seen, in all cases upon the oil usage, the concentrations are reduced continuously. This means that upon the oil application, depletion in additives is occurred. In fact, at high engine temperature, the additives are degraded and some of the resulting degradation products are absorbed by the filter [38]. which result in lowering of the concentrations of the corresponding elements in the oil. It is interesting to note that the amount of reduction is more sever for zinc and Phosphorus, which indicate that depletion of corresponding additives are more than the other ones.

 Wear metals will appear in the oil because of wearing of different parts of engine, Fe is the most common of the wear metals. Present in some form in virtually all equipment. Its widespread presence means that there are many sources of the wear particles. It can be found in cylinder liners, piston rings, valve train, crankshaft, rocker arms, spring gears, lock washers, nuts, pins, connecting rods, engine blocks, and oil pump. Cu is widely used as an alloying element, copper is prized because of its materials properties, very ductile and excellent thermal and electrical conductivity. It is heavily used in bearing systems, as well as heat exchangers. In the engine, it can be found in valve train bushing, wrist pin bushing, cam bushings, oil cooler core, thrust washers, governor, connecting rods bearings, and valve gear train thrust buttons. Tin is used as an alloying element with copper and lead for sacrificial bearing liners. In the engine, it can be found in valve train bushing, wrist pin bushing, cam bushings, oil cooler core, thrust washers, governor, connecting rods bearings, and valve gear train thrust buttons. Aluminum is valued in equipment because of it high strength to weight ratio and excellent corrosion resistance. Being alloyed with other elements improves its wear and temperature resistance. It is widely specified for equipment manufacture nowadays. In engine, it can be found in engine blocks, pistons, blowers, oil pump bushings, bearings (some), cam bushings (some), and oil coolers (some). Chromium is used as an engineering material for its great hardness and corrosion resistance. It is found in many systems operating under harsh conditions. In engine it can be found in rings, liners, exhaust valves, and zinc chromate from cooling system inhibitor. Lead is used in a soft metal used for sacrificial wear surfaces such as journal bearings. Lead-based babbitts are widely used. Silver has exceptional thermal conductivity and is an excellent bearing plate material, providing minimum friction. It is susceptible to corrosive attack by zinc-based additives. In engine, it can be found in valves, valve guides, cylinder liners, and bearings. The other elements can also find in different parts of engine [2].

 The data in Table 1 indicate that, upon increase of running kilometer, the concentration of wear elements has been increased continuously. Boron is an exclusion. This means that during the performance, some wear has been occurred in different parts of the engine. Among the elements, the most wear belongs to iron and manganese. As it can be seen, the concentration of iron has been changed 12 units and the concentration of manganese has been changed 15.5 units. Meanwhile, the concentration change of other elements has not been changed considerably. Thus, more wear has occurred for the corresponding equipment.

 The decreasing trend of boron concentration may be due to the formation of boron compounds in oil matrix, which are absorb in oil filters.

 The concentrations of the elements, which are neither due to base oil, nor due to additive, are given in Table 1. As it can be seen, the concentrations of wear elements that are, manganese, iron, copper, tin, titanium, vanadium, lead, cadmium, and antimony have been increased. Meanwhile, the concentration of potassium, which is a contaminant element [3] show a sudden increase in 11500 km. This may be due to initiation of some leakage of coolant to the oil. Alternatively, the water absorption may be the source of this increase.

 Consistency, flow properties, or viscosity in the case of oils are key parameters to create lubrication efficiency and the application of lubricants [39]. The viscosity of used engine oil can drop for reasons of fuel dilution or because of high water content and/or shearing of the VI improver [3]. Viscosity can increase because of heavy contamination of the oil by soot, polymerization, vaporization losses, and emulsions due to water contamination and/or oxidation of the oil [3]. Obviously, the final status of the oil viscosity depends on the combination effects of decreasing and increasing factors. If the falling factors are overcome to the rising ones, the drop in viscosity will happen. An increase in the property will be observed, in the reverse conditions.

**Figure 1:**
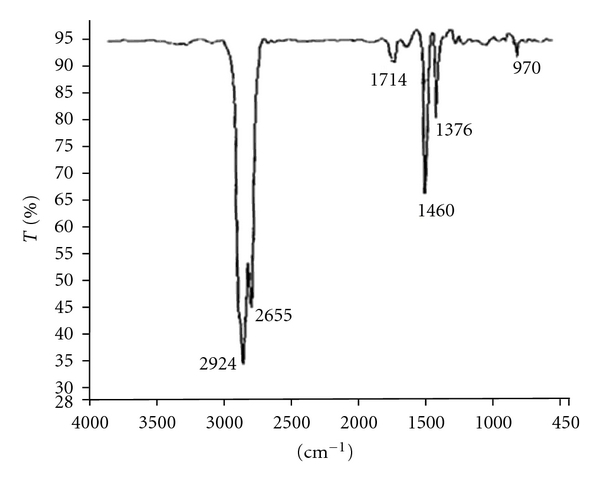
IR spectrum of the oil after 11500 km operation.

 As it can be seen from the Table 3 until 2000 km running, the viscosity at 40°C and 100°C decreases systematically. After 2000 km the reverse trend is observed. This indicates that before 2000 km, the viscosity decreasing factors such as fuel dilution, water contamination, and shearing of the VI improver conquer to increasing ones. Meanwhile, at running kilometers more than 2000, the increasing factors such as heavy contamination of the oil by soot, polymerization, vaporization losses, and emulsions due to water contamination and/or oxidation of the oil are overcome to decreasing agents. Since there is no signal of fuel dilution, decreasing of viscosity can mainly attributed to water contamination and shearing of the VI improver. On the other hand, absence of 2000 cm^−1^ band in IR spectrum of used oil (Figure 1) which is an important sign of soot formation [18, 19], as well as, lack of satisfactory reasons for vaporization losses and formation of emulsion, verify that polymerization and oxidation are the major motivation of viscosity increase. 

The observed bands in the IR spectrum of used oil (Figure 1) can be assigned to CH stretching (2924 cm^−1^), carbonyl (1714 cm^−1^), CH_2_ scissoring (1460 cm^−1^), symmetric bending of CH_3_ (1376 cm^−1^), and aromatic compounds (970 cm^−1^). The observation of recent bands can be attributed to the existence of aromatic, naphthenic, and aliphatic compounds, which are constituents of employed mineral base oil (SN-500). In addition, the observation of carbonyl band indicates that some oxidation has been occurred. The low intensity of the observed peak means that the degree of oxidation is low.

 The flash point is the lowest temperature at which an ignition source causes the vapors of the specimen (lubricant) to ignite under specified conditions [40]. Like viscosity, the flash point test has always been a standard part of a lubricant's specification. Because of the low-flash points of most fuels, a sudden drop in flash temperature in crankcase oil can usually be relied upon as an indication of dilution. Occasionally, very high, localized temperatures can lead to thermal cracking within the oil. As no variation of flash point is observed both thermal cracking and fuel dilution are discarded. The fixation of pour point, which is the normal result of thermal cracking, is a further confirmation of the absence of thermal cracking.

 Fuel dilution causes the decrease the specific gravity. In contrast, silicon contamination or oxidation causes its increase [2]. If both of increasing and decreasing factors exist simultaneously, the final situation will be determined by the preference factor. Observation of increasing trend (Table 1), beside the absence of fuel dilution or contamination indicates that oxidation is the main reason of the raise in specific gravity.

 The total acid number is a measure of the acidic constituents in petroleum products. The acidity of unused oils and fluids is normally derived from the type and concentration of specific additive material, whereas the acidity of used oil is of interest to measure the degree of oxidation of the fluid. The total base number (TBN) characterizes the alkaline reserve in petroleum products [34]. It is particularly used for engine oils, where by acidic combustion products use up the alkaline reserve. Both TAN and TBN can be obtained by acid base titration.

**Figure 2:**
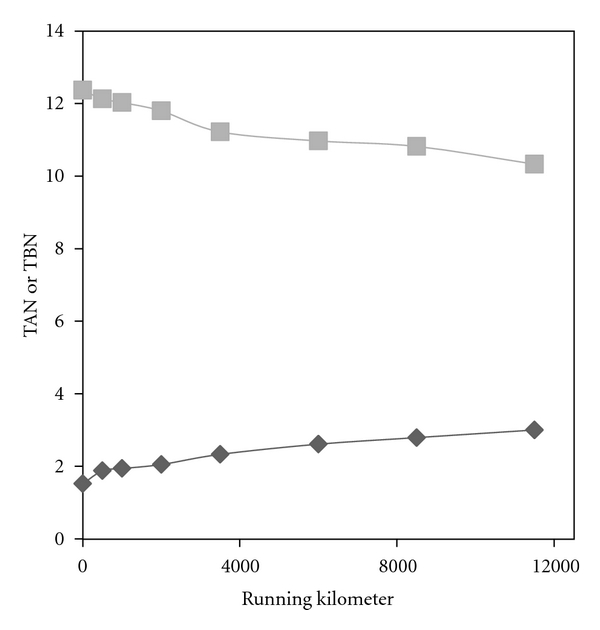
Plots of TAN (bottom) or TBN (top) versus running kilometers.

 The plots of TAN and TBN versus kilometers of operation are shown in Figure 2 as it can be seen upon increasing the running kilometer, the TAN increases continuously. The rising trend of TAN can be attributed to oxidation of some of the lubricant constituents and subsequent formation of carboxylic acids. In fact, upon increasing the operation time of the oil, the antioxidant additives will deplete gradually. The depletion of antioxidants beside the high temperature of engine and presence of oxygen provides suitable circumstances for oxidation. The appearance of carbonyl band in IR spectrum of used oil (Figure 1) is a further evidence of oxidation.

 In contrast, to TAN a lessening drift is observed for TBN (Figure 2). This decreasing trend can be assigned to depletion of additives, which mostly do have basic character. This is in accordance with the results of elemental analysis (Table 1) which fairly confirms the depletion of additives.

 Considering that, TBN is a measure of alkaline reservation of lubricant [29]. It is expected that after complete consumption of alkaline materials the neutralization to be stopped completely and sudden elevation of TAN to be observed. Obviously, after this point, the destructive effects of acidic products will be very high and further usage of the oil is not wisdom. Upon extrapolation of TBN and TAN curves, it can be predicted that approximately at 23000 km these two recent values will be equal. Therefore, it can be concluded that 23000 km is a critical value and the time of oil replacing.

 Water is the most common contaminant found in lubricating oils. It is also one of the most damaging to bearings and other lubricated components. It causes corrosion to metal surfaces, lubricant degradation, and poor lubrication. Water can be present in three forms of dissolved, emulsified, and free in lubricating oils. The concentration of dissolved water is less than 100 ppm and is not harmful nor does it affect the appearance or performance of the lubricant. Emulsified water exists in more than 150 ppm and causes milky appearance of the oil. It is the most harmful. Water droplets are the third kind of water in lubricating oils. This form of water in oil is also very harmful to lubricated parts but is also the easiest to separate [3]. As it can be seen from the Table 3, despite increasing trend of water content, its concentration has not reached to critical value. Consequently, in present oil, water cannot be regarded as an important harmful factor and its contribution in damages is negligible.

## 4. Conclusion

 Based on the observed results it can be concluded that through different operation kilometers the following changes are happened:

1. the additives depletion;
2. minor wear;
3. some oxidation;
4. increase and decrease in rheological properties;
5. increase of TAN and decrease of TBN due to oxidation;
6. a few water contaminations;
7. not many coolant leakage.

In addition, the soot formation and fuel contamination do not happen.

## Figures and Tables

**Table 1:** The concentration of elements containing in additives, at different distances. Values in parenthesis are due to base oil.

No.	Element	Kilometer of operation							
		0	500	1000	2000	3500	6000	8500	11500
(1)	S (7000)	1108.3	970.4	964.7	957.5	960.0	940.0	935.4	907.1
(2)	Zn (6.1)	784.0	743.2	711.6	650.1	580.9	467.5	355.6	249.9
(3)	P (5.7)	811.2	793.9	773.5	738.0	686.6	603.5	505.1	411.9
(4)	Mg (0.3)	228.9	228.3	227.6	225.4	223.2	222.1	214.7	202.7
(5)	Si (3.1)	61.4	60.9	60.1	59.1	59.1	51.5	50.7	50.1
(6)	Ca (<DL)	56.7	53.5	51.9	46.9	43.7	36.4	29.4	22.3
(7)	Ba (<DL)	29.4	29.1	28.4	27.8	26.9	23.8	23.7	23.4
(8)	B (6.5)	6.7	6.2	5.7	5.3	4.8	3.8	3.5	3.3
(9)	Mo (6.4)	6.5	6.5	6.5	6.6	7.5	8.2	8.6	8.8
(10)	Al (5.2)	5.1	5.2	5.4	5.9	5.9	7.0	7.3	8.9
(11)	Ag (1.6)	1.7	2.1	2.2	2.2	2.3	2.2	2.3	2.3
(12)	Cr (1.1)	1.1	1.6	1.9	2.1	2.2	2.3	2.4	2.7
(13)	Ni (1.2)	1.1	1.2	1.3	1.4	1.6	1.6	1.7	2.0
(14)	Na (0.4)	0.5	0.7	0.8	1.0	1.1	1.0	1.2	1.2
(15)	Mn (<DL)	<DL	0.8	1.1	1.4	2.6	3.2	4.5	15.5
(16)	Fe (<DL)	<DL	2.4	3.7	5.2	6.9	8.6	10.1	11.8
(17)	Cu (<DL)	<DL	0.4	0.8	1.2	1.8	1.8	2.1	2.5
(18)	Sn (<DL)	<DL	0.4	0.8	0.8	0.9	1.0	1.3	1.4
(19)	Ti (<DL)	<DL	0.4	0.8	1.1	1.8	1.8	1.9	1.9
(20)	V (<DL)	<DL	0.5	0.7	1.3	1.8	1.8	1.8	1.9
(21)	Pb (<DL)	<DL	<DL	<DL	<DL	0.1	0.2	0.2	0.6
(22)	Cd (<DL)	<DL	0.5	0.5	0.5	0.6	0.6	0.6	0.6
(23)	Sb (<DL)	<DL	0.3	0.5	0.6	0.7	0.7	0.8	0.8
(24)	K (<DL)	<DL	<DL	<DL	<DL	<DL	<DL	<DL	7.4

**Table 2:** Standard deviations of the data of Table 1.

No.	Element	Kilometer of operation							
		0	500	1000	2000	3500	6000	8500	11500
(1)	S (±0.3)	±0.3	±0.5	±0.2	±0.1	±0.1	±0.3	±0.7	±0.3
(2)	Zn (±0.9)	±0.1	±0.1	±0.5	±0.8	±0.6	±0.3	±0.2	±0.1
(3)	P (±0.8)	±0.3	±0.3	±0.3	±0.3	±0.3	±0.3	±0.3	±0.3
(4)	Mg (±0.6)	±0.1	±0.1	±0.1	±0.9	±0.4	±0.6	±0.3	±0.5
(5)	Si (±0.6)	±0.4	±0.1	±0.7	±0.5	±0.2	±0.2	±0.6	±0.1
(6)	Ca (—)	±0.3	±0.7	±0.6	±0.3	±0.5	±0.9	±0.1	±0.3
(7)	Ba (—)	±0.3	±0.3	±0.3	±0.3	±0.3	±0.3	±0.3	±0.3
(8)	B (±0.1)	±0.6	±0.1	±0.1	±0.7	±0.8	±0.1	±0.5	±0.9
(9)	Mo (±0.3)	±0.1	±0.1	±0.4	±0.2	±0.1	±0.1	±0.3	±0.3
(10)	Al (±0.3)	±0.3	±0.6	±0.3	±0.7	±0.1	±0.3	±0.4	±0.5
(11)	Ag (±0.1)	±0.5	±0.2	±0.1	±0.5	±0.6	±0.5	±0.4	±0.5
(12)	Cr (±0.1)	±0.1	±0.3	±0.4	±0.5	±0.6	±0.6	±0.3	±0.1
(13)	Ni (±0.1)	±0.3	±0.2	±0.3	±0.4	±0.4	±0.3	±0.3	±0.9
(14)	Na (±0.2)	±0.1	±0.1	±0.2	±0.2	±0.1	±0.2	±0.2	±0.3
(15)	Mn (—)	—	±0.2	±0.1	±0.3	±0.1	±0.1	±0.1	±0.5
(16)	Fe (—)	—	±0.2	±0.5	±0.3	±0.3	±0.6	±0.1	±0.2
(17)	Cu (—)	—	±0.1	±0.2	±0.6	±0.5	±0.1	±0.2	±0.2
(18)	Sn (—)	—	±0.2	±0.1	±0.1	±0.3	±0.1	±0.2	±0.3
(19)	Ti (—)	—	±0.5	±0.7	±0.4	±0.2	±0.1	±0.1	±0.3
(20)	V (—)	—	±0.3	±0.1	±0.4	±0.2	±0.1	±0.1	±0.3
(21)	Pb (—)	—	—	—	—	±0.1	±0.1	±0.1	±0.2
(22)	Cd (—)	—	±0.1	±0.1	±0.1	±0.2	±0.2	±0.2	±0.3
(23)	Sb (—)	—	±0.1	±0.1	±0.1	±0.3	±0.4	±0.1	±0.2
(24)	K (—)	—	—	—	—	—	—	—	±0.3

**Table 3:** Physical properties at different running kilometer.

Property	Test method	Running Kilometer							
		0	500	1000	2000	3500	6000	8500	11500
Viscosity at 40°C	ASTM D-445	141.6	140.0	138.3	135.3	137.2	137.8	142.2	143.4
Viscosity at 100°C	ASTM D-445	16.5	16.3	16.0	15.8	15.9	16.1	16.3	16.5
Viscosity index	ASTM D-2270	125.0	126.2	127.3	129.5	128.0	127.5	123.0	122.2
Flash point	ASTM D-92	222	—	—	—	—	—	—	223
Pour point	ASTM D-97	−26	—	—	—	—	—	—	−26
Specific gravity	ASTM D-1298	0.8910	0.8935	0.8942	0.8943	0.8950	0.8963	0.8994	0.9011
Color	ASTM D-1500	2.0	2.8	3.1	3.9	5.1	5.9	6.3	7.5
TAN (mg KOH/g)	ASTM D-664	1.52	1.88	1.94	2.05	2.33	2.61	2.79	3.00
TBN (mg KOH/g)	ASTM D-664	12.37	12.13	12.03	11.80	11.22	10.97	10.82	10.33
Water content	ASTM D-6304	22.1	35.2	43.0	50.1	54.9	61.4	63.0	63.0

**Table 4:** 

Element	Wavelength	Element	Wavelength	Element	Wavelength	Element	Wavelength
S (10.0)	181.6	Ba (0.03)	233.5	Ni (0.5)	231.6	Ti (0.4)	334.9
Zn (0.2)	206.2	B (1.0)	249.7	Na (0.5)	589.6	V (0.5)	290.9
P (4.0)	213.6	Mo (0.5)	202.1	Mn (0.1)	257.6	Pb (1.0)	230.3
Mg (0.04)	285.2	Al (1.0)	396.1	Fe (0.1)	238.2	Cd (0.1)	328.8
Si (10.0)	251.6	Cr (0.2)	267.7	Cu (0.4)	327.4	Sb (2.0)	206.8
Ca (0.05)	317.9	Ag (0.6.0)	328.1	Sn (2.0)	189.9	K (1.0)	766.5
