# Expression of Concern on “Monitoring of the Physical and Chemical Properties of a Gasoline Engine Oil during Its Usage”

**DOI:** 10.1155/2019/5024873

**Published:** 2019-02-03

**Authors:** Journal of Analytical Methods in Chemistry

*Journal of Analytical Methods in Chemistry* would like to express concern with the article titled “Monitoring of the Physical and Chemical Properties of a Gasoline Engine Oil during Its Usage” published in *Journal of Analytical Methods in Chemistry* in March 2012 [1] due to a claim of data fabrication.

The journal received a claim that the data in this article were falsified or fabricated. We asked the corresponding author, Dr. Semnani, to provide documentation of the output for the data and spectra, but he said that the original documents have been discarded though the data is correct. A member of the journal's editorial board confirmed that there were concerns about the article and the author's response, and we therefore asked the author's institution, the University of Shahrekord, to investigate. We have been in contact with the Investigating Committee for Ethics and Research, but the investigation has not yet concluded. Due to the delay, the journal's editors would like to inform readers of concerns about the integrity of the data in this article.

Update, June 2020:

The journal has received correspondence from the author's institution that the investigation has concluded and no misconduct was identified. Full details of the investigation were not provided to the journal for verification and the editorial board are not satisfied that the concerns have been resolved.
